# Exosomal microRNAs as tumor markers in epithelial ovarian cancer

**DOI:** 10.1002/1878-0261.12371

**Published:** 2018-10-09

**Authors:** Chi Pan, Ines Stevic, Volkmar Müller, Qingtao Ni, Leticia Oliveira‐Ferrer, Klaus Pantel, Heidi Schwarzenbach

**Affiliations:** ^1^ Department of Tumor Biology University Medical Center Hamburg‐Eppendorf Germany; ^2^ Department of Gynecology University Medical Center Hamburg‐Eppendorf Germany

**Keywords:** apoptosis, cell proliferation, epithelial ovarian cancer, exosomes, ovarian cystadenoma, prognosis

## Abstract

Specific microRNAs (miRNAs) are packaged in exosomes that regulate processes in tumor development and progression. The current study focuses on the influence of exosomal miRNAs in the pathogenesis of epithelial ovarian cancer (EOC). MiRNA profiles were determined in exosomes from plasma of 106 EOC patients, eight ovarian cystadenoma patients, and 29 healthy women by TaqMan real‐time PCR‐based miRNA array cards containing 48 different miRNAs. In cell culture experiments, the impact of miR‐200b and miR‐320 was determined on proliferation and apoptosis of ovarian cancer cell lines. We report that miR‐21 (*P* = 0.0001), miR‐100 (*P* = 0.034), miR‐200b (*P* = 0.008), and miR‐320 (*P* = 0.034) are significantly enriched, whereas miR‐16 (*P* = 0.009), miR‐93 (*P* = 0.014), miR‐126 (*P* = 0.012), and miR‐223 (*P* = 0.029) are underrepresented in exosomes from plasma of EOC patients as compared to those of healthy women. The levels of exosomal miR‐23a (*P* = 0.009, 0.008) and miR‐92a (*P* = 009, 0.034) were lower in ovarian cystadenoma patients than in EOC patients and healthy women, respectively. The exosomal levels of miR‐200b correlated with the tumor marker CA125 (*P* = 0.002) and patient overall survival (*P* = 0.019). MiR‐200b influenced cell proliferation (*P* = 0.0001) and apoptosis (*P* < 0.008). Our findings reveal specific exosomal miRNA patterns in EOC and ovarian cystadenoma patients, which are indicative of a role of these miRNAs in the pathogenesis of EOC.

**Abbreviations**
EOC: epithelial ovarian cancer
FIGO: Federation of Gynecology and Obstetrics
miRNA: microRNA
ROC: receiver operating characteristic

## 1 Introduction

In the female reproductive system, epithelial ovarian cancer (EOC) is one of the most aggressive carcinomas and the leading cause of death among gynecologic malignancies due to its frequent recurrence. EOC develops asymptomatically at early stages, so the disease is rarely detected, which might contribute to the poor prognosis. More than two‐thirds of patients are diagnosed at advanced FIGO stages (III or IV) and have a 5‐year survival rate of less than 40%. In contrast, patients who are diagnosed with FIGO stage I or II have a higher 5‐year survival rate of 70–90% (Jayson *et al*., 2014). Current diagnostic methods for detection and monitoring of EOC mainly include pelvic examination, transvaginal ultrasound, and measurement of the serum biomarker CA125 (carbohydrate antigen 125). In particular, the determination of CA125 is not sufficiently specific to diagnose EOC at an early stage, since its values are only elevated in approximately 50% of stage I, but in 70–90% of advanced disease (Felder *et al*., 2014). Therefore, new diagnostic biomarkers are urgently needed, and such markers could be exosomal microRNAs (miRNAs). In addition, the biology of EOC is not sufficiently understood, since some patients respond to chemotherapy while others get primary resistance.

Exosomes are microvesicles ranging from approximately 30–100 nm in size and are secreted by all living cells (Simpson *et al*., 2009). Cancer‐derived exosomes may mediate propagation of cancer by transferring pathogenic factors from cancer to healthy cells (Chen *et al*., 2012; Valadi *et al*., 2007). Thus, discharging of the exosome contents in the recipient cells can modulate the fate of the recipient cells. Since exosomes can cover wide distances, they can influence distantly located cells and tissues. Increased secretion of exosomes has been associated with tumor invasiveness both *in vitro* and *in vivo* and to promote migration and proliferation of tumor cells leading to metastasis (Azmi *et al*., 2013). In a previous study, we detected high concentrations of exosomes in the serum of EOC patients (Meng *et al*., 2016). This excessive, active exosome secretion prompted us to intensify our study on exosomes along with their cargo in EOC patients.

Apart from protein, lipids, DNA, and RNA, the cargo of exosomes also includes miRNAs. The process of sorting and packaging of miRNAs into exosomes seems to be selective, favoring certain miRNAs for exosomal transfer over others (Pant *et al*., 2012; Schwarzenbach, 2015). Of clinical relevance is thereby which miRNAs are increasingly incorporated into exosomes in the different tumor stages. MiRNAs are a family of evolutionary conserved, small noncoding RNA molecules consisting of approximately 22 nucleotides. As one of the largest gene families, miRNAs account for approximately 1% of the human genome and are highly conserved in nearly all organisms (Kim, 2005). They inhibit gene expression post‐transcriptionally by binding specifically to the 3′ untranslated region (3′ UTR) of their target mRNA. Gene silencing occurs through translational inhibition or cleavage of their target mRNA. Both processes depend on complementary sequences in the binding sites of the specific miRNA and target mRNA (Bartel, 2009). Computational analyses indicate that one miRNA has a binding affinity to hundreds of different mRNAs, and hence, miRNAs regulate numerous signal transduction pathways, for example, involved in cell proliferation and differentiation, as well as development and progression of benign and malignant diseases (Cheng *et al*., 2005). In mammals, they are believed to regulate approximately 50% of all protein‐coding genes (Krol *et al*., 2010). In particular, miRNAs incorporated in exosomes and participating in cell‐to‐cell communication may play a crucial role in EOC development, progression, and metastasis.

In the present study, we deepened our search for clinically relevant exosomal miRNAs in EOC. Using miRNA array cards containing 48 different miRNAs, we analyzed the enrichment of miRNAs in exosomes and found a set of eight exosomal miRNAs in EOC and two exosomal miRNAs in ovarian cystadenoma patients that were deregulated compared with healthy women. Quantification of exosomal miR‐200b was of diagnostic and prognostic relevance. In cell culture experiments, this miRNA inhibited cell proliferation and promoted apoptosis.

## 2 Materials and methods

### 2.1 Study populations

**Figure 1:**
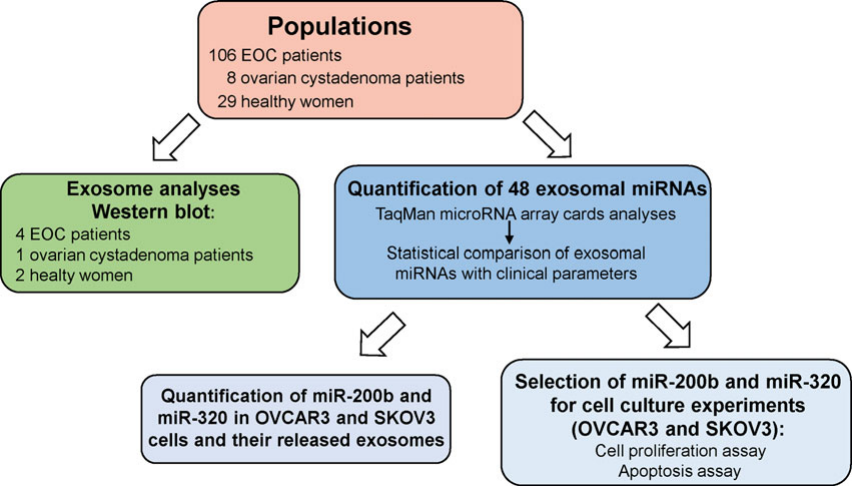
Workflow of the study.

Blood samples were collected from 106 EOC patients directly before surgery from September 2009 to October 2015. The patients were treated according to national guidelines at the University Medical Center Hamburg‐Eppendorf, Department of Gynecology, and histologically confirmed for International Federation of Gynecology and Obstetrics (FIGO) stages I–IV. The median follow‐up time was 22 months (range from 1 to 247 months). Detailed patient characteristics are summarized in Table 1. In addition, the present study includes plasma samples from eight ovarian cystadenoma patients and 29 healthy women who had no history of cancer. Blood samples of ovarian cystadenoma patients were obtained from May 2017 to June 2017, while those of healthy women were collected during 2015 and 2016. Mean ages of EOC patients, ovarian cystadenoma patients, and healthy women were 60, 61, and 56 years and ranged from 28 to 81, from 45 to 81, and from 47 to 69 years, respectively. Blood collection and experiments were performed in compliance with the Helsinki Declaration and were approved by the ethics committee (Ethik‐Kommission der Ärztekammer, Hamburg, Germany, PV5392). The experiments were undertaken with the understanding and written informed consent of each subject. Regarding blood processing, uniform management concerning the specific protocols was performed. Sample flow is described in Fig. 1.

**Table 1:** EOC patient characteristics

Ovarian cancer patients (%)	106 (100)
Age (mean)	60 (28–81 years)
Follow‐up time (median)	22 (1–247 months)
Recurrence (%)	
Yes	49 (46.2)
No	57 (53.8)
Histology (%)	
Serous	90 (84.9)
Other subtypes	13 (12.3)
Unknown	3 (2.8)
FIGO stage (%)	
I–III	72 (67.9)
IV	20 (18.9)
Unknown	14 (13.2)
Grading (%)	
G1–2	25 (23.6)
G3	72 (67.9)
Unknown	9 (8.5)
Lymph node (%)	
N0	17 (16.1)
N1	56 (52.8)
Unknown	33 (31.1)
Secondary carcinoma (%)	
Yes	18 (17.0)
No	88 (83.0)
Tumor residual (%)	
Tumor‐free	61 (57.5)
Tumor rest	38 (35.8)
Unknown	7 (6.6)
Disease‐free survival (months)	17 (1–84)
CA 125 U·mL^−1^ (%)	
<65	5 (4.7)
≥65	69 (65.1)
Unknown	32 (30.2)
Survival status (%)	
Dead	35 (33.0)
Alive	71 (67.0)

### 2.2 Verification of hemolysis in plasma samples

To avoid quantifying exosomal miRNA in hemolytic plasma samples that may influence our results, we performed hemoglobin measurements by spectral analysis (Kirschner *et al*., 2011). Red blood cells were lysed in 7 mL whole blood by erythrocyte lysis buffer (containing 0.3 m sucrose, 10 mm Tris pH 7.5, 5 mm MgCl_2_, and 1% Triton X‐100). A dilution series (1 : 1, 1 : 3, 1 : 4, 1 : 6, 1 : 8, 1 : 10, 1 : 12, 1 : 14, 1 : 18, 1 : 20) of lysed red blood cells was prepared in plasma and served as a standard curve for the measurement of hemolysis in all plasma samples. Fifty microliters of each plasma sample was measured in duplicates on a Microplate reader (Tecan, Männerdorf, Switzerland). Absorbance peaks at 414, 541, and 576 nm were indicative for free hemoglobin, with the highest peak at 414 nm. The higher the absorbance in plasma samples is, the higher is the degree of hemolysis. The average values and standard deviations were calculated from the duplicates (Fig. S1).

### 2.3 Isolation of total exosomes from plasma and cell lines

Plasma was prepared by three centrifugation steps at 300 *g* for 10 min, 2000 *g* for 10 min, and 10 000 *g* for 30 min to remove cells and cell debris. Exosomes were then isolated from 143 plasma samples as well as from supernatants of ovarian cancer cell lines OVCAR3 and SKOV3 by ExoQuick (BioCat, Heidelberg, Germany) according to the manufacturer's instructions. Briefly, 500 μL of plasma and 5 mL cell supernatants were incubated with 120 μL and 1 mL ExoQuick exosome precipitation solution, respectively, at 4 °C for 30 min. Then, continued with a centrifugation at 1500 *g* for 30 min to remove the supernatants and additional 5 min to remove residual fluid. The precipitated exosomes were resuspended in PBS (phosphate‐buffered saline) buffer (Life Technologies, Darmstadt, Germany).

### 2.4 Western blot

To calculate the adequate protein amounts for carrying out a western blot, the protein concentrations were measured with the DC Protein Assay Kit (Bio‐Rad, Munich, Germany) at a wavelength of 650 nm on a spectrophotometric plate reader (Tecan). A standard curve of 0, 0.625, 1.25, 2.5, 5, and 10 mg·mL^−1^ bovine serum albumin (Sigma‐Aldrich Chemie, Munich, Germany) was applied by the double‐dilution method. Three microliters of exosomes and standard protein samples were added to 96‐well plates according to the manufacturer's instructions. The protein concentrations were calculated according to a linear equation of *y* = *m*x + *n* by applying the linear regression method. Thirty micrograms of exosomes were electrophoretically separated and blotted onto a poly(vinylidene difluoride) membrane (Millipore, Billerica, MA, USA) which was subsequently incubated with antibodies specific for the exosomal marker CD63 (ABGENT, San Diego, CA, USA) and the miRNA binding protein AGO2 (TAKARA Bio Inc., Shiga, Japan) overnight. Detection of the proteins was carried out using a peroxidase‐conjugated secondary antibody (Dako, Glostrup, Denmark) and a chemiluminescence ECL detection solution (Sigma‐Aldrich, St. Louis, MO, USA).

### 2.5 Extraction of exosomal miRNAs and conversion into cDNA

MiRNAs were extracted from exosomes and cell lines resuspended in 150 μL lysis buffer by using the TaqMan miRNA ABC Purification Bead kit Human panel A (Thermo Fisher Scientific, Darmstadt, Germany) according to the manufacturer's recommendations. For extraction efficiency, 2 μL of 1 nm synthetic cel‐miR‐39 was added as an exogenous spike in control. The extracted miRNAs were immediately reverse‐transcribed into cDNA using a modified protocol of TaqMan MicroRNA Reverse Transcription kit (Thermo Fisher Scientific). For miRNA quantification in plasma‐derived exosomes, the 15 μL reaction contained 6 μL Custom RT primer pool, while for miRNA quantification in cell lines and cell‐derived exosomes, the reaction contained 6.0 μL TaqMan RT primers of miR‐484, cel‐miR‐39, miR‐200b, and miR‐320 diluted in 1 : 100 Tris–EDTA. Both reactions were supplemented with 0.3 μL 100 mm dNTPs including dTTP, 3 μL 50 U·μL^−1^ MultiScribe reverse transcriptase, 1.5 μL 10× RT buffer, 0.19 μL 20 U·μL^−1^ RNase inhibitor (Thermo Fisher Scientific), and 4 μL RNA, and were carried out at 16 °C for 30 min, 42 °C for 30 min, and 85 °C for 5 min on a MJ Research PTC‐200 Peltier Thermal Cycler (Global Medical Instrumentation, Ramsey, MN, USA).

### 2.6 Preamplification of exosomal miRNAs

To increase the input cDNA, a preamplification step of cDNA was included. For miRNA quantification in plasma‐derived exosomes, 5 μL of cDNA was preamplified in a 25 μL reaction containing 3.75 μL Custom PreAmp Primer Pool and 12.5 μL TaqMan PreAmp Master Mix (Thermo Fisher Scientific). For miRNA quantification in cell lines and cell‐derived exosomes, 1.5 μL of cDNA was preamplified in a 15 μL reaction containing 2.25 μL PreAmp primer pool of miR‐484, cel‐miR‐39, miR‐200b, and miR‐320 diluted in 1 : 100 Tris–EDTA and 7.5 μL TaqMan PreAmp Master Mix. PCR was run on a MJ Research PTC‐200 Peltier Thermal Cycler (Global Medical Instrumentation): one cycle at 95 °C for 10 min, 55 °C for 2 min, 72 °C for 2 min; 16 cycles at 95 °C for 15 s, 60 °C for 4 min; and a terminal cycle at 99.9 °C for 10 min. To avoid false‐positive data (e.g., primer dimer formation or unspecific PCR products), a negative control without any templates was included from the starting point of reverse transcription.

### 2.7 Exosomal miRNA expression profiling

Custom TaqMan microRNA Array Cards (Thermo Fisher Scientific), quantitative real‐time PCR‐based cards used for miRNA expression profiling, contain assays for the detection of 44 human miRNAs of interest, two endogenous reference miRNAs (RNU6, miR‐484), one exogenous reference miRNA (cel‐miR‐39), and one N/A‐4343438‐blank (negative control). We selected the 44 miRNAs because they have been described to be clinically relevant for cancer in the literature and in our previous studies (Meng *et al*., 2015, 2016; Roth *et al*., 2011; Schwarzenbach, 2016). These miRNAs of interest were then mounted on miRNA array cards (Thermo Fisher Scientific) and are as follows: let7g, miR‐9, miR‐16, miR‐20a, miR‐21, miR‐23a, miR‐23b, miR‐26a, miR‐27a, miR‐27b, miR‐29a, miR‐30c, miR‐34c‐5p, miR‐92a, miR‐93, miR‐100, miR‐103, miR‐126, miR‐141, miR‐152, miR‐182, miR‐187, miR‐191‐5p, miR‐200a, miR‐200b, miR‐200c, miR‐203, miR‐205, miR‐214, miR‐221, miR‐223, miR‐320a, miR‐373, miR‐422a, miR‐429, miR‐485‐5p, miR‐496, miR‐519a, miR‐520 g, miR‐548a‐3p, miR‐574‐3p, miR‐590‐5p, miR‐625‐5p, miR‐891a.

To carry out real‐time TaqMan PCR for miRNA quantification in plasma‐derived exosomes, the protocol of Thermo Fisher Scientific was modified as followed: a 112.5‐μL PCR was prepared with 56.25 μL TaqMan Universal Master Mix II and 2 μL preamplification product and loaded on the array cards. PCR was run on a 7900 HT Fast Real‐Time PCR System (Applied Biosystems, Foster City, CA, USA): one cycle at 95 °C for 10 min and 40 cycles at 95 °C for 15 s, 60 °C for 1 min.

For miRNA quantification in cell lines and cell‐derived exosomes, in a 20 μL reaction, 0.5 μL preamplified cDNA was mixed with 10 μL TaqMan Universal PCR Master Mix and 1 μL TaqMan MicroRNA Assay. Quantitative real‐time PCR was performed at 95 °C for 10 min, and in 40 cycles at 95 °C for 15 s and at 60 °C for 60 s, on a C1000 Touch real‐time PCR device (Bio‐Rad, Hercules, CA, USA).

### 2.8 Data normalization and statistical analyses

The statistical analyses were performed using the thermo fisher scientific analysis Software, Relative Quantification Analysis Module, version 3.1 (http://www.aps.thermofisher.com), and spss software package, version 22.0 (SPSS Inc., Chicago, IL, USA). First, the obtained data of the miRNA expression levels were calculated and evaluated by the ΔCq method as follows: ΔCq = mean value Cq (reference miR‐484) − mean value Cq (miRNA of interest). For data normalization, snRNU6 could not be used as a reference because of its too instable expression, whereas the expression of miR‐484 remained relatively constant across the plasma samples. The interindividual variability was controlled by spiking of cel‐miR‐39 and was low.

The thermo fisher scientific analysis Software was used for performing hierarchical clustering (heat map) and volcano plots. Distances between samples and assays were calculated for hierarchical clustering based on the ΔCq values using Pearson's correlation. Clustering method was average linkage. Subsequently, the relative expression data were 2^ΔCq^ transformed in order to obtain normal distribution data. The confidence of 2^ΔCq^ data was verified by amplification curves and Cq confidence (0–1, whereby 1 refers to the highest confidence). Our data showed a Cq confidence of 0.95. Values below 0.95 were discarded.

Statistical differences of exosomal miRNA expressions between healthy controls, ovarian cystadenoma patients, and EOC patients were calculated using two‐tailed Student's *t*‐test and depicted as a volcano plot. Diagnostic power of the exosomal miRNAs was analyzed by receiver operating characteristic (ROC) curves. The correlations of plasma levels of exosomal miRNAs with clinical parameters were calculated by using ANOVA Tukey's HSD test and Spearman rho test. To estimate overall and disease‐free survival, log‐rank test and Kaplan–Meier plots were carried out. Missing data were handled by pairwise deletion. A *P*‐value <0.05 was considered as statistically significant.

### 2.9 Cell culture, miRNA quantification, and transient transfection

OVCAR3 and SKOV3 cells were purchased from ATCC (American Type Culture Collection) and authenticated by the Leibniz Institute DSMZ (Deutsche Sammlung von Mikroorganismen und Zellkulturen GmbH), Braunschweig, on 30 July 2013 and 14 June 2013, respectively. Aliquots were frozen in liquid nitrogen, and a new aliquot of these cell lines was used for this study. OVCAR3 and SKOV3 cells were cultured in RPMI 1640‐ and McCoy's 5A‐modified medium, respectively. For the MTT and apoptosis assays, the media were supplemented with 10% FBS (PAA, Laboratories, Cölbe, Germany), while for quantification of cell‐derived miRNAs and exosomes, they were supplemented with 10% exosome‐depleted FBS (Norgen Biotech, Thorold, ON, Canada). Cells were cultured under standard conditions (37 °C, 5% CO_2_, humidified atmosphere) and regularly tested for mycoplasma contamination (Minerva Biolabs, Berlin, Germany).

To detect transfection efficiency, both cell lines transfected with miScript miRNA mimic miR‐200b or miR‐320 at final concentrations of 10 nm, together with 15 μL HiPerFect^®^ Transfection Reagent (Qiagen, Hilden, Germany), were harvested after 24 h and lysed in 50 μL PBS and 150 μL lysis buffer for extraction of miRNAs as described above.

### 2.10 MTT assay

OVCAR3 and SKOV3 cells were seeded into 96‐well plates at a density of 8000 and 4000 cells per well in triplicate and transfected with double‐stranded miScript miRNA mimic or single‐stranded miScript inhibitor of miR‐200b and miR‐320 or AllStars‐negative control small interfering RNA (negative control) at final concentrations of 10, 50, and 10 nm, respectively, together with 0.75 μL HiPerFect^®^ Transfection Reagent (Qiagen). Following 24‐, 48‐, and 72‐h transfection with miRNA mimics, inhibitors, or negative control, cells were incubated with 20 μL 5 mg·mL^−1^ MTT (thiazolyl blue tetrazolium bromide; Sigma‐Aldrich, USA) in PBS at 37 °C for 3 h. Then, the cells were lysed with lysis buffer (4 mm HCl, 0.1% NP40 in isopropanol), to solubilize the colored crystals. OD (optical density) was measured at 540 nm on a microplate reader (Tecan). Each experiment contained three replicate wells and was repeated three times.

### 2.11 Apoptosis assay

OVCAR3 and SKOV3 cells were seeded into six‐well plates at density of 150 000 and 100 000 cells per well in triplicate. After 24 h, cells were transfected with miRNA mimics, inhibitors, or negative control. Cells were treated with 25 μm topoisomerase I inhibitor camptothecin for 4 h to induce apoptosis. To determine camptothecin‐ and miRNA‐mediated apoptosis, the FITC Annexin‐V Apoptosis Detection Kit I (BD Pharmingen, San Diego, CA, USA) was used according to the manufacturer's instructions. The cell apoptosis was measured on a FACS Canto II flow cytometer (BD Biosciences).

## 3 Results

### 3.1 Workflow

Using real‐time PCR‐based miRNA array cards, 44 miRNAs (plus four references) were quantified in exosomes derived from plasma samples of 106 EOC patients, eight ovarian cystadenoma patients, and 29 healthy women. Exosomes were quantified by a western blot. Following the quantification of exosomal miRNAs, the array data were normalized by the exogenous cel‐miR‐39 and an endogenous reference miRNA. We selected miR‐484 as a reference miRNA because they displayed constant values through the cohorts of EOC patients, ovarian cystadenoma patients, and healthy women. The normalized data were statistically evaluated and compared with the clinical parameters of EOC patients. In addition, we selected miR‐200b and miR‐320 for further *in vitro* and functional analyses because they were significantly deregulated in exosomes from plasma of EOC patients, and their dual character as tumor suppressor genes and oncomiRs with multiple cancer‐specific functions have been described in diverse studies (Li *et al*., 2017; Meng *et al*., 2015; Muralidhar and Barbolina, 2015; Wang *et al*., 2017). We quantified the levels of these two miRNAs in two cell lines (OVCAR3 and SKOV3) and in exosomes released from these cells. The impact of the significantly deregulated miR‐200b and miR‐320 on cell proliferation and apoptosis was also analyzed by overexpression of these miRNA. Figure 1 summarizes the single steps of the workflow.

### 3.2 Verification of exosomes

**Figure 2:**
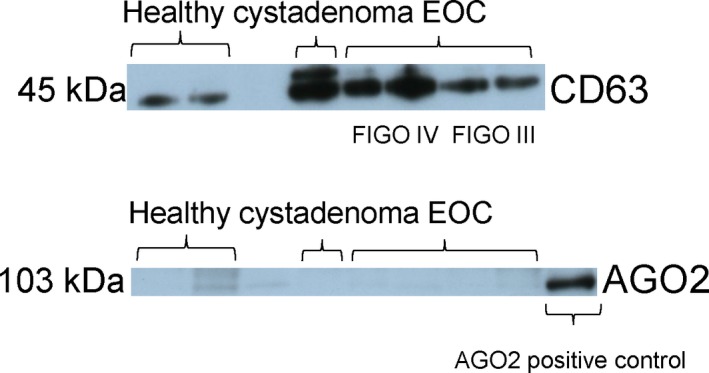
Verification and quantification of exosomes. Exosomes were precipitated from plasma of healthy women, ovarian cystadenoma patients, and EOC patients by the agglutinating agent ExoQuick and analyzed by western blots using antibodies specific for the exosome proteins CD63 and the miRNA‐associated AGO2 protein. The western blots show representative examples of exosomes, devoid of cell‐free miRNAs.

Prior to quantification of exosomal miRNAs, extracted exosomes from two healthy women, one ovarian cystadenoma patient, and each two EOC patients at FIGO stage III and stage IV were verified on a western blot using antibodies specific for the exosomal marker CD63 and the miRNA binding protein AGO2. As shown by the 45‐kDa band on the blot, the CD63‐specific antibody recognized nonlysed exosomes in the pellet. Strikingly, the exosome bands were stronger in the cystadenoma patient and EOC patients at FIGO stage IV than in healthy women and EOC patients at FIGO stage III. As shown by the 103‐kDa band on the blot, the AGO2‐specific antibody did not detect AGO2 protein bound to cell‐free miRNAs in the exosome pellet (Fig. 2). These findings show that the exosome fraction may be pure and devoid of cell‐free miRNAs. However, they do not exclude that exosomes may contain traces of contaminations of cell‐free AGO2‐bound miRNAs that due to the low sensitivity of the western blot were not detectable.

### 3.3 Different miRNA signatures in exosomes of EOC patients and ovarian cystadenoma patients

Next, we carried out a quantitative TaqMan real‐time PCR using microRNA array cards containing 48 different miRNAs, to determine miRNA expression profiles in exosomes derived from the plasma of 106 EOC patients, eight ovarian cystadenoma patients, and 29 healthy women (Fig. 1). We selected miRNAs for the assembly of the 48‐microarray cards due to their oncogenic/tumor suppressive function in EOC, as described in the literature (PubMed) and in our previous studies (Meng *et al*., 2015, 2016; Roth *et al*., 2011; Schwarzenbach, 2016). The selected miRNAs are listed in Materials and methods. Then, a similarity matrix was generated containing all pairwise similarities of the plasma samples of EOC patients, ovarian cystadenoma patients, and healthy controls. To detect potential clusters in rows (miRNAs) and columns (plasma samples) of the normalized expression matrix, hierarchical clustering was performed. The relative up‐ and downregulated miRNA are indicated by red and green, respectively. Clustering analysis confirmed the observed difference in gene expression patterns between the three groups. For example, 19 of 29 samples (~ 66%) form a cluster exclusively comprised of samples from healthy women, 100 of 106 samples (~ 94%) form a cluster exclusively comprised of EOC samples, and four of eight samples (~ 50%) form a cluster exclusively comprised of samples from ovarian cystadenoma. Thus, 123 of 143 samples (~ 86%) are located within the expected clusters (heat map, Fig. S2).

**Figure 3:**
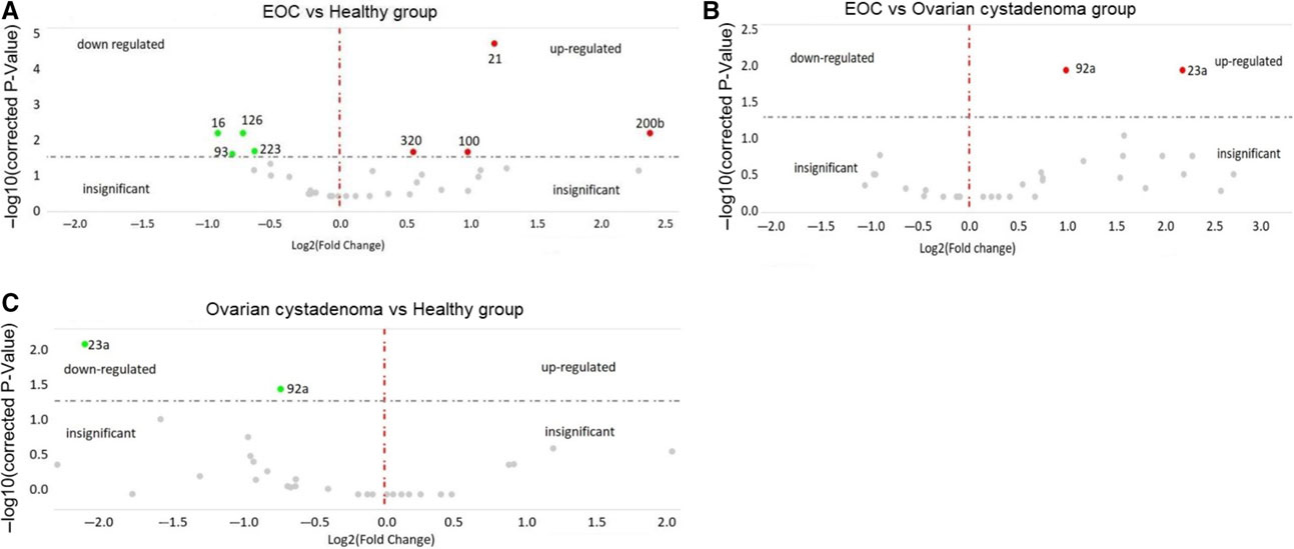
Volcano plots of 45 exosomal miRNAs. The plots were drawn for comparison of exosomal miRNAs in plasma of 106 EOC patients with those of 29 healthy women (A) and eight ovarian cystadenoma patients (B) as well as between ovarian cystadenoma patients and healthy women (C). The log2 fold changes are plotted on the *x*‐axis, and the negative log10 *P*‐values are plotted on the *y*‐axis. The left side shows downregulated exosomal miRNAs (green dots). The right side shows upregulated exosomal miRNAs (red dots). Under the dashed horizontal line, there are nonderegulated miRNAs (gray dots).

To compare the enrichment of miRNAs in exosomes derived from plasma of 106 EOC patients with that of 29 healthy women (A), 106 EOC patients with that of eight ovarian cystadenoma patients (B), and eight ovarian cystadenoma patients with that of 29 healthy women (C), we drew volcano plots (Fig. 3). Table 2 summarizes the significant results with the adjusted *P*‐values and fold changes of miRNAs as derived from the volcano plots (Fig. 3). From 44 miRNAs, four miRNAs (miR‐21, miR‐100, miR‐200b, and miR‐320) were significantly enriched, whereas four miRNAs (miR‐16, miR‐93, miR‐126, and miR‐223) were underrepresented in exosomes of EOC patients compared with those of healthy women. The levels of exosomal miR‐23a and miR‐92a were significantly lower in plasma of ovarian cystadenoma patients than in healthy women and EOC patients, but similar in healthy women and EOC patients, indicating that both miRNAs play rather a role in benign ovarian tumors (Fig. 3, Table 2).

**Table 2:** *P*‐values with the corresponding fold changes of exosomal miRNAs in bold

Populations	Patient No.			miR‐16	miR‐21	miR‐23a	miR‐92a	miR‐93	miR‐100	miR‐126	miR‐200b	miR‐200c	miR‐223	miR‐320
EOC vs healthy^a^	106 vs 29		Fold change	**0.5**	**2.3**	1.0	1.2	**0.5**	**2.0**	**0.6**	**5.2**	4.9	**0.6**	**1.5**
			*P*‐value	**0.009**	**0.0001**	1.000	0.148	**0.014**	**0.034**	**0.012**	**0.008**	0.143	**0.029**	**0.034**
EOC vs cystadenoma^a^	106 vs 8		Fold change	0.9	3.0	**4.6**	**2.0**	0.9	12.4	0.5	2.3	3.5	0.7	1.5
			*P*‐value	1.00	0.100	**0.009**	**0.009**	1.000	0.210	0.210	0.261	0.725	0.976	0.630
Cystadenoma vs healthy^a^	8 vs 29		Fold change	0.6	0.8	**0.2**	**0.6**	0.6	0.2	1.1	2.3	1.4	0.9	1.0
			*P*‐value	0.478	0.839	**0.008**	**0.034**	0.770	0.177	1.000	0.229	1.000	1.000	1.000
Clinical parameters in EOC patients														
Recurrence^a^	Yes	48	Fold change	**0.5**	1.1	1.0	0.8	0.6	1.4	1.0	1.3	1.1	0.8	0.9
	No	54	*P*‐value	**0.001**	0.648	0.845	0.073	0.05	0.224	0.780	0.710	0.841	0.366	0.717
Histology^a^	Serous	90	Fold change	0.7	**0.5**	0.7	**0.7**	0.7	0.6	0.7	0.7	0.6	0.7	0.7
	Others	13	*P*‐value	0.432	**0.026**	0.285	**0.010**	0.413	0.192	0.162	0.686	0.289	0.177	0.190
Grading^a^	G1–2	24	Fold change	**0.5**	0.8	1.0	0.8	0.7	1.3	1.0	4.1	0.8	1.2	0.8
	G3	69	*P*‐value	**0.019**	0.504	0.995	0.150	0.200	0.341	0.946	0.144	0.524	0.366	0.226
CA125^b^			*P*‐value	0.325	0.377	0.637	0.124	0.415	0.156	0.762	**0.002**	**0.003**	0.835	0.363

^a^ *P*‐values are calculated by two‐tailed Student's *t*‐test. ^b^ *P*‐values are calculated by bivariate analyses of Spearman rho test.

**Figure 4:**
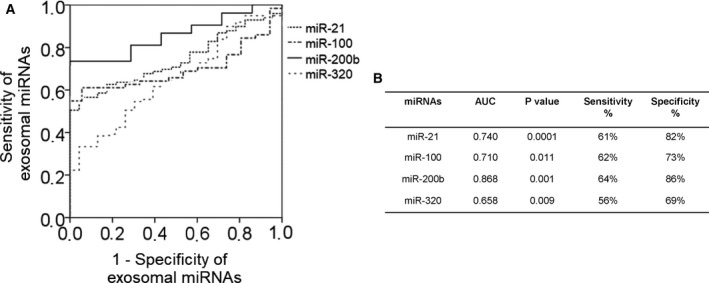
Exosomal miRNAs differ between EOC patients and healthy women. ROC analyses (A) show the profiles of sensitivity and specificity of exosomal miR‐21, miR‐100, miR‐200b, and miR‐320, to distinguish EOC patients from healthy women. The table (B) summarizes sensitivities and specificities of exosomal miR‐21, miR‐100, miR‐200b, and miR‐320.

In addition, we performed ROC analyses of the most deregulated miRNAs. The significant differences of exosomal miRNA concentrations between EOC patients and healthy women were reflected by the AUC values of miR‐21 (0.740), miR‐100 (0.710), miR‐200b (0.868), and miR‐320 (0.658). We also calculated the sensitivity and specificity of these exosomal miRNAs by the highest Youden index. The levels of exosomal miR‐21, miR‐100, and miR‐200b could best discriminate between EOC patients and healthy women with a sensitivity of 61%, 62%, and 64% and a specificity of 82%, 73%, and 86% (Fig. 4). The combination of the concentrations of exosomal miR‐21, miR‐100 and miR‐200b could not increase the sensitivity and specificity (data not shown).

### 3.4 Correlation to risk factors and survival

**Figure 5:**
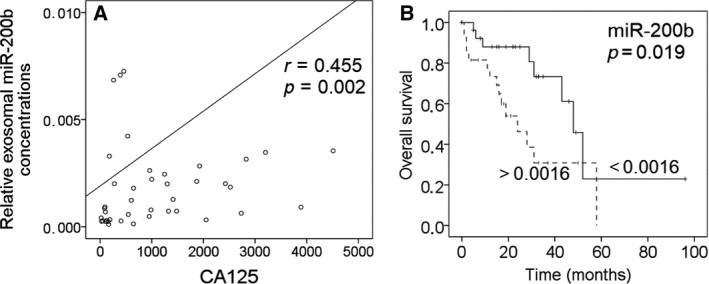
Association of exosomal miR‐200b with the tumor marker CA125 and prognosis. The scatter plot shows the correlation of increasing concentrations of exosomal miR‐200b with increasing CA125 values (A). The univariate Kaplan–Meier curve shows the correlation of low and high levels of exosomal miR‐200b with overall survival. The median values of the exosomal miRNA concentration (0.0016) were used for grouping the EOC samples according to low and high transcript levels (B).

Table 2 includes the significant correlations of the levels of exosomal miR‐16, miR‐21, miR‐92a, miR‐200b, and miR‐200c with the clinicopathological/risk parameters of EOC patients. Lower levels of exosomal miR‐16 were significantly associated with recurrence (*P* = 0.001) and grading 1–2 (*P* = 0.019). Strikingly, the levels of miR‐16 were lower in exosomes from EOC patients than healthy women (*P* = 0.009), and they still decreased in recurrent patients compared with healthy women (*P* = 0.002). The concentrations of miR‐21 (*P* = 0.026) and miR‐92a (*P* = 0.010) were lower in exosomes in patients with serious EOC than patients with other histological subtypes (Table 2). Moreover, the plasma levels of exosomal miR‐200b were significantly associated with increasing values of the tumor marker CA125 (*P* = 0.002, Fig. 5A, Table 2). Also, the levels of exosomal miR‐200c significantly correlated with increasing CA125 values (*P* = 0.003, Table 2). However, the levels of miR‐200c were not significantly deregulated in EOC patients, although they were 4.9‐fold upregulated in EOC patients compared with healthy women (*P* = 0.143, Table 2). Since no CA125 values were measured in ovarian cystadenoma patients and healthy women, we could not compare and combine its sensitivity and specificity with those of exosomal miR‐200b and miR‐200c to detect EOC.

To assess the prognostic potential of the plasma levels of exosomal miRNAs in EOC patients, uni‐ and multivariate logistic regression models for overall and disease‐free survival were carried out. The median follow‐up time was 22 months (range from 1 to 247 months). In Kaplan–Meier and log‐rank models, EOC patients were grouped due to higher and lower levels of exosomal miRNAs than the median miRNA values. In univariate analysis, the higher concentrations of exosomal miR‐200b were associated with poor overall survival (*P* = 0.019, Fig. 5B), but not in multivariate analysis with the covariable nodal status (*P* = 0.631). Besides, a positive nodal status correlated with poor overall survival (*P* = 0.017, data not shown).

### 3.5 MiR‐200b inhibits cell proliferation and promotes apoptosis

We selected miR‐200b and miR‐320 for functional analysis, because they were significantly deregulated in exosomes from EOC patients, and their dual character as tumor suppressor genes and oncomiRs with multiple cancer‐specific functions have been described in diverse studies (Li *et al*., 2017; Meng *et al*., 2015; Muralidhar and Barbolina, 2015; Wang *et al*., 2017). At first, we measured the basal levels of miR‐200b and miR‐320 in ovarian cancer cell lines OVCAR3 and SKOV3 and in their released exosomes. With the exception of the expression of miR‐200b which was hardly detectable in SKOV3 cells, both miRNAs were expressed in the cell lines. Their levels were usually higher in the cell‐derived exosomes than in the cells, except for the expression levels of miR‐200b in exosomes from OVCAR3 cells (Fig. S3).

**Figure 6:**
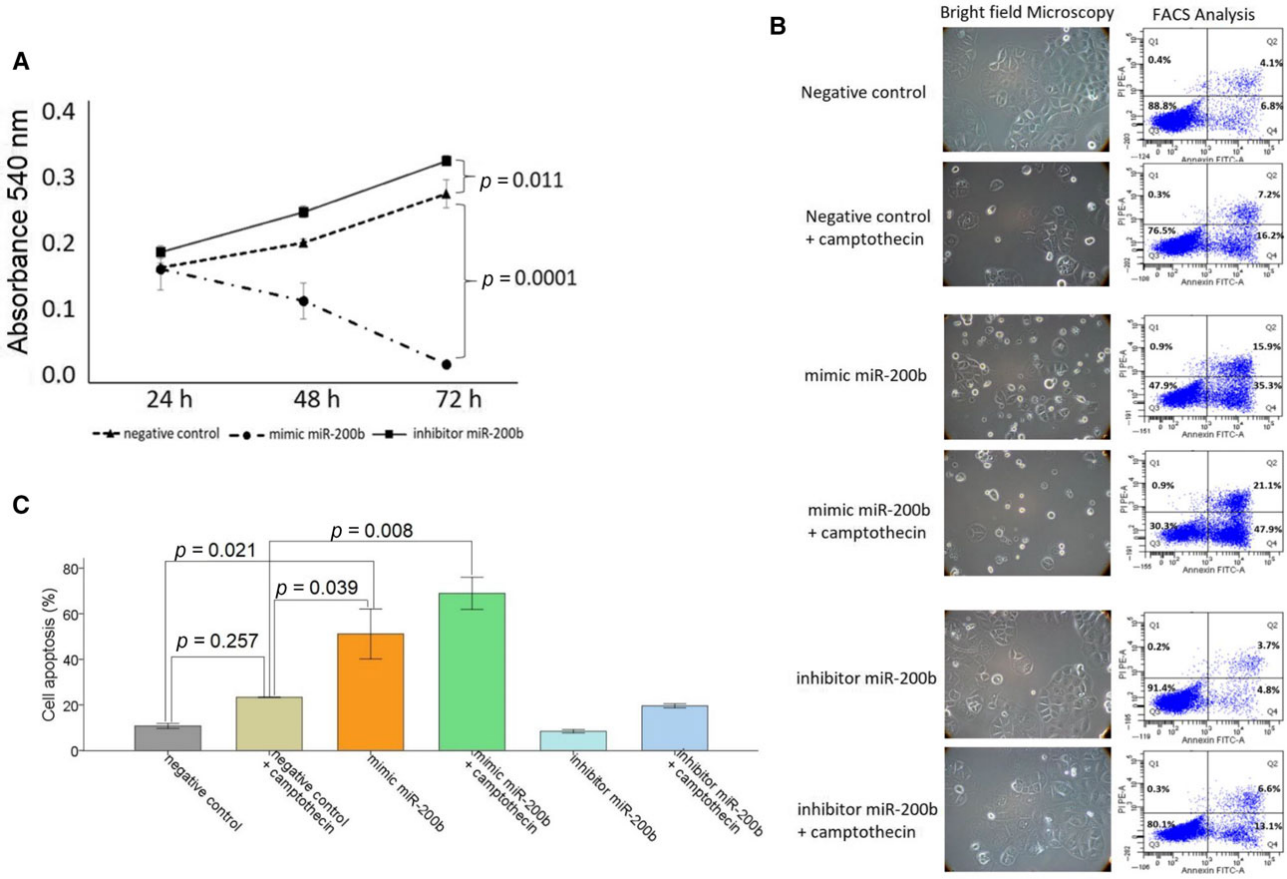
MiR‐200b affects cell proliferation and apoptosis. OVCAR3 cells were transiently transfected with a negative control, mimic, or inhibitor of miR‐200b. After 24, 48, and 72 h, cells were treated with MTT. Cell proliferation after overexpression and inhibition of miR‐200b was measured at an absorbance of 540 nm. The standard deviations from triplicate experiments are indicated in the line chart (A). OVCAR3 cells were additionally treated with the topoisomerase I inhibitor camptothecin and analyzed by a bright‐field microscopy (left) and on a FACS Canto II device (right) for apoptosis. In order to observe morphological features of apoptosis, cells were analyzed using 10× magnification. Cells were labeled with Annexin‐V‐FITC and propidium iodide for FACS analyses. Cell fragments only positive for propidium iodide can be found in the upper left corner (Q1). Late apoptotic as well as necrotic cells can be found in the upper right corner (Q2), since they are positive for Annexin and propidium iodide. Living cells are negative for Annexin and propidium iodide and therefore can be found in the lower left corner (Q3). Early apoptotic cells are only positive for Annexin and located in the lower right corner (Q4). The size for each population (%) is given in the corresponding area (B). A bar chart summarizes the data on the apoptotic effect by miR‐200b as derived from the FACS CantoII device. Three independent experiments were performed. Error bars are presented as means ± SD. ANOVA Tukey's HSD tests were used to determine the significance (*P* < 0.05) (C).

Then, the effects of these miRNAs on cell proliferation and apoptosis were investigated in OVCAR3 and SKOV3 cells which were transiently transfected with mimics and inhibitors of miR‐200b and miR‐320. Before performing the functional analyses, we measured the transfection efficiency. The levels of miR‐200b in transfected cell lines OVCAR3 and SKOV3 were about 71‐ and 103‐fold higher than in untransfected cell lines, respectively. Moreover, the levels of miR‐320 in transfected cell lines OVCAR3 and SKOV3 were about 27‐ and 11‐fold higher than in untransfected cell lines, respectively. In Fig. 6, the results for miR‐200b in OVCAR3 cells are depicted. Transfection with mimics and inhibitors of miR‐200b steadily reduced (*P* = 0.0001) and increased (*P* = 0.011) cell proliferation in OVCAR3 cells during 72 h, respectively, compared with the negative control, indicating that miR‐200b inhibits cell proliferation (Fig. 6A).

For the apoptosis assay, the transfected cells were additionally treated with topoisomerase I inhibitor camptothecin. Camptothecin is used in cancer chemotherapy to induce apoptosis. As expected, bright‐field images and FACS analyses showed that camptothecin and miR‐200b mediated apoptosis (Fig. 6B,C). In OVCAR3 cells, the apoptotic effect by camptothecin was not significant (*P* = 0.257), but could be increased by the additional administration of miR‐200b mimics (*P* = 0.008). Moreover, miR‐200b could also induce apoptosis by itself, and this effect was stronger than the effect by camptothecin (*P* = 0.021). Inhibition of miR‐200b had only a weak and nonsignificant effect on apoptosis (Fig. 6B,C).

In SKOV3 cells, miR‐200b did not affect cell proliferation and apoptosis (Fig. S4), although the cells displayed the highest transfection efficiency with miR‐200b. Moreover, the transfections with mimics and inhibitors of miR‐320 had no effect on proliferation and apoptosis in both cell lines (Fig. S5).

## 4 Discussion

In the present study, we analyzed the enrichment of miRNAs in exosomes derived from plasma of EOC patients, ovarian cystadenoma patients, and healthy women by a quantitative TaqMan real‐time PCR using microRNA array cards. We found that out of 44 miRNAs, eight miRNAs (miR‐16, miR‐21, miR‐93, miR‐100, miR‐126, miR‐200b, miR‐223 and miR‐320) were deregulated in exosomes from EOC patients compared with healthy women. The levels of exosomal miR‐23a and miR‐92a were associated with ovarian cystadenoma patients. In addition, we detected higher plasma levels of exosomes in ovarian cystadenoma and EOC patients, in particular in FIGO IV patients, than in healthy women and FIGO III patients. The low levels of exosomes found in healthy women may be mainly derived from platelets, erythrocytes, and endothelial cells and have been shown to be important in common hemostatic events in normal physiology (Yuana *et al*., 2013). The higher levels of exosomes in FIGO IV than in FIGO III patients suggest that a higher release of exosomes correlates with their higher invasive potential. In line to our observations, Kobayashi *et al*. (2014) reported that high invasive ovarian cancer cells release significantly more exosomes than low‐invasive cells. This high secretion of exosomes that are important mediators between tumor and its microenvironment may contribute to increase cell invasion, form a premetastatic niche, and prepare secondary sites for metastasis (Steinbichler *et al*., 2017). Surprisingly, we also detected high plasma levels of exosomes in ovarian cystadenoma patients, suggesting that an excessive secretion of exosomes also occurs in these tumors reflecting extensive inflammatory processes that are not malignant.

Besides the increased secretion of exosomes, the selective packaging of miRNA may contribute to the pathogenesis of ovarian tumors. As far as we know, we demonstrate for the first time sets of enriched miR‐21, miR‐100, miR‐200b, and miR‐320 as well as of underrepresented miR‐16, miR‐93, miR‐126, and miR‐223 in exosomes of EOC patients compared with those of healthy women. In our study, we found the highest increase in concentrations of miR‐200b in exosomes from EOC patients compared with those in exosomes of healthy women. The levels of this exosomal miRNA were about five times higher in EOC patients than in healthy women and correlated with the tumor marker CA125 currently routinely used as screening tests for EOC, and with poor overall survival in EOC patients. In comparison with the other exosomal miRNAs analyzed, miR‐200b had also the highest sensitivity (64%) and specificity (86%) to discriminate EOC from healthy women. Our present data substantiate our previous findings also showing the positive relationship of exosomal miR‐200b with CA125 values and its prognostic relevance in EOC patients (Meng *et al*., 2016). On the other hand, our *in vitro* experiments showed that miR‐200b acts rather as a tumor suppressor, inhibiting cell proliferation and promoting apoptosis of ovarian cancer cells, confirming previously reported data (Zuberi *et al*., 2015). However, miR‐200 family members seem to be versatile players (Muralidhar and Barbolina, 2015). They play a major role in the suppression of epithelial–mesenchymal transition and metastasis (Burk *et al*., 2008; Koutsaki *et al*., 2014), and low‐level expression of miR‐200 members in advanced ovarian tumors significantly correlated with cancer recurrence and poor overall survival, whereas overexpression of miR‐200b inhibited ovarian cancer cell migration (Zuberi *et al*., 2015). In contrast, other studies have shown that its elevated expression is a significant characteristic of EOC tissue and serum (Kan *et al*., 2012; Liu *et al*., 2012). Moreover, using microRNA array card analysis, the levels of miR‐200b derived from EpCAM‐positive exosomes were reported to be higher in EOC patients than in patients with benign ovarian diseases (Taylor and Gercel‐Taylor, 2008). These findings demonstrate the enrichment of miR‐200b in exosomes and its dual character in EOC.

Likewise, miR‐320 seems also to have a dual character. It was reported that high expression of miR‐320 is associated with negative prognosis, migration, and invasion of cancer and high risk of metastasis in EOC (Wang *et al*., 2017). In contrast, another study revealed that miR‐320 is rather a tumor suppressor due to its downregulation in EOC tissue compared with nontumor tissue, and its ability to suppress cell proliferation, cycle, and invasion through targeting Twist homolog 1 in EOC. In our study, we found that miR‐320 is preferentially packaged in exosomes from EOC patients, but has no impact on cell proliferation and apoptosis in ovarian cancer cell lines.

The first direct evidence of miRNAs playing a role in human cancer came from Calin *et al*. (2002). They demonstrated that the miRNA cluster containing miR‐16 was deleted in a significant portion of chronic lymphocytic leukemia cases (Calin *et al*., 2002). To date, miR‐16 has been used as a reference miRNA for data normalization due to its steadily high expression and, conversely, has been reported to act as tumor suppressor or oncomiR in different cancer types, thus to be involved in inhibiting and promoting cancer development and progression both *in vitro* and *in vivo* (Huang *et al*., 2015; Schwarzenbach, 2016; Schwarzenbach *et al*., 2014; Stückrath *et al*., 2015). MiR‐16 may also be a potential therapeutic target and clinical biomarker of bone metastasis, because it is elevated in osteoclast differentiation and bone metastasis (Ell *et al*., 2013). In our present study, we found that lower amounts of miR‐16 were packaged in exosomes from EOC patients, while in our previous study, we detected that the serum levels of circulating miR‐16 were neither up‐ nor downregulated in EOC patients compared with healthy women (Meng *et al*., 2015). Moreover, we observed that the decreased levels of miR‐16 in exosomes from EOC patients further decreased in recurrent patients, suggesting that decrease in levels of exosomal miRNAs is particularly associated with recurrence. An association of miR‐16 with both ovarian cancer survival and recurrence were previously reported for tumor tissues (Delfino and Rodriguez‐Zas, 2013).

MiR‐100 can exert both tumor suppressor and oncogenic functions in various cancer types, too. However, in EOC, miR‐100 seems to be a tumor suppressor, since its levels are usually lower in EOC tissues than in adjacent normal tissues (Peng *et al*., 2012). Upregulation of miR‐100 can inhibit cell proliferation, promote cell apoptosis and cell cycle arrest, and sensitize resistant EOC cells to cisplatin, resulting in reversing drug resistance (Guo *et al*., 2016). In our present study, we show that the levels of miR‐100 were two times higher in exosomes from EOC patients than those in healthy women, indicating a selective packaging process and a potential occurrence of exosomes whose cargo also contains tumor suppressive potential.

MiR‐21 is one of the best‐studied miRNA, is overexpressed in most cancer types and, thus, displays oncogenic activity. Accumulating evidence supports a central role for miR‐21 in ovarian cancer initiation, progression, and chemoresistance (Schwarzenbach *et al*., 2014). Here, we show that high amounts of miR‐21 were also packaged in exosomes from EOC patients. MiR‐93 was described to be a potential suppressor of ovarian cell proliferation and to inhibit EOC tumorigenesis and progression by targeting the small G protein/guanosine triphosphatase RhoC (Chen *et al*., 2015). MiR‐126 is also a tumor suppressor in EOC and decreases serine/threonine p21‐activated kinase 4 expression to inhibit invasive growth of EOC cells (Luo *et al*., 2015). In line with the tumor suppressive behavior of these two miRNA, our quantitative analyses show an approximately twice lower presence of miR‐93 and miR‐126 in exosomes from EOC patients than in healthy women. Aberrant miR‐223 expression has been implicated in the pathogenesis of a wide range of cancers. In EOC, miR‐223 seems to serve as an oncomiRs (Haneklaus *et al*., 2013). However, we found a downregulation of this miRNA in exosomes from EOC patients compared with healthy women, thus a decrease in its oncogenic potential in exosomes. These findings demonstrate that the extent of enrichment of miRNAs in exosomes does not reflect tumor suppressive or oncogenic functions of miRNAs. For example, Huang *et al*. (2017) showed a significant overexpression of miR‐223 in serum from esophageal squamous cell carcinoma patients compared with normal controls, but similar levels of this miRNA in exosomes from both cohorts.

Finally, our analyses showed lower levels of exosomal miR‐23a and miR‐92a in ovarian cystadenoma patients than in healthy women. A deregulation of miR‐23a could not be observed in exosomes from EOC patients and a downregulation of miR‐92a only in exosomes from serious EOC patients but not in patients harboring a different histology. Our findings point to a role of these miRNAs in benign ovarian tumors, in particular of miR‐23a. A recent study by Xiong *et al*. also showed that the levels of serum miR‐23a were significantly lower in women with polycystic ovary syndrome (PCOS) than healthy women. The likelihood of women with PCOS decreased by 0.01‐fold for every onefold increase in miR‐23a expression (Xiong *et al*., 2017).

## 5 Conclusion

In conclusion, our findings suggest a specific miRNA pattern in exosomes from EOC and ovarian cystadenoma patients. The frequent dual character of miRNA in tumor cells is accompanied by the enrichment of oncogenic and tumor suppressive miRNAs in exosomes that participate in cell‐to‐cell communication. Continuing studies are required to reveal the complex regulatory network of expression and packaging of miRNAs into exosomes that participate in the pathogenesis of EOC.

## Author contributions

HS made the conception and design. HS, CP, and IS participated in the development of methodology. CP performed the experiments. VM and LOF contributed to the acquisition of samples and clinical data. CP, QN, and IS analyzed the data. HS, CP, and KP wrote, reviewed, and/or revised the manuscript. All authors read and approved the final manuscript.

## Supporting information


**Fig. S1.** Levels of free hemoglobin measured in the plasma samples.

**Fig. S2.** Hierarchical cluster of 48 exosomal miRNAs.

**Fig. S3.** miR‐200b and miR‐320 levels in cell lines and their released exosomes.

**Fig. S4.** miR‐200b does not affect cell proliferation and apoptosis in SKOV3 cells.

**Fig. S5.** miR‐320 does not affect cell proliferation and apoptosis.
