# Supplementary figures and images for: Exosomal microRNAs as tumor markers in epithelial ovarian cancer
Source: Mol Oncol. 2018 Oct 9;12(11):1935–48. doi: 10.1002/1878-0261.12371 (PMC6210043; doi:10.1002/1878-0261.12371)

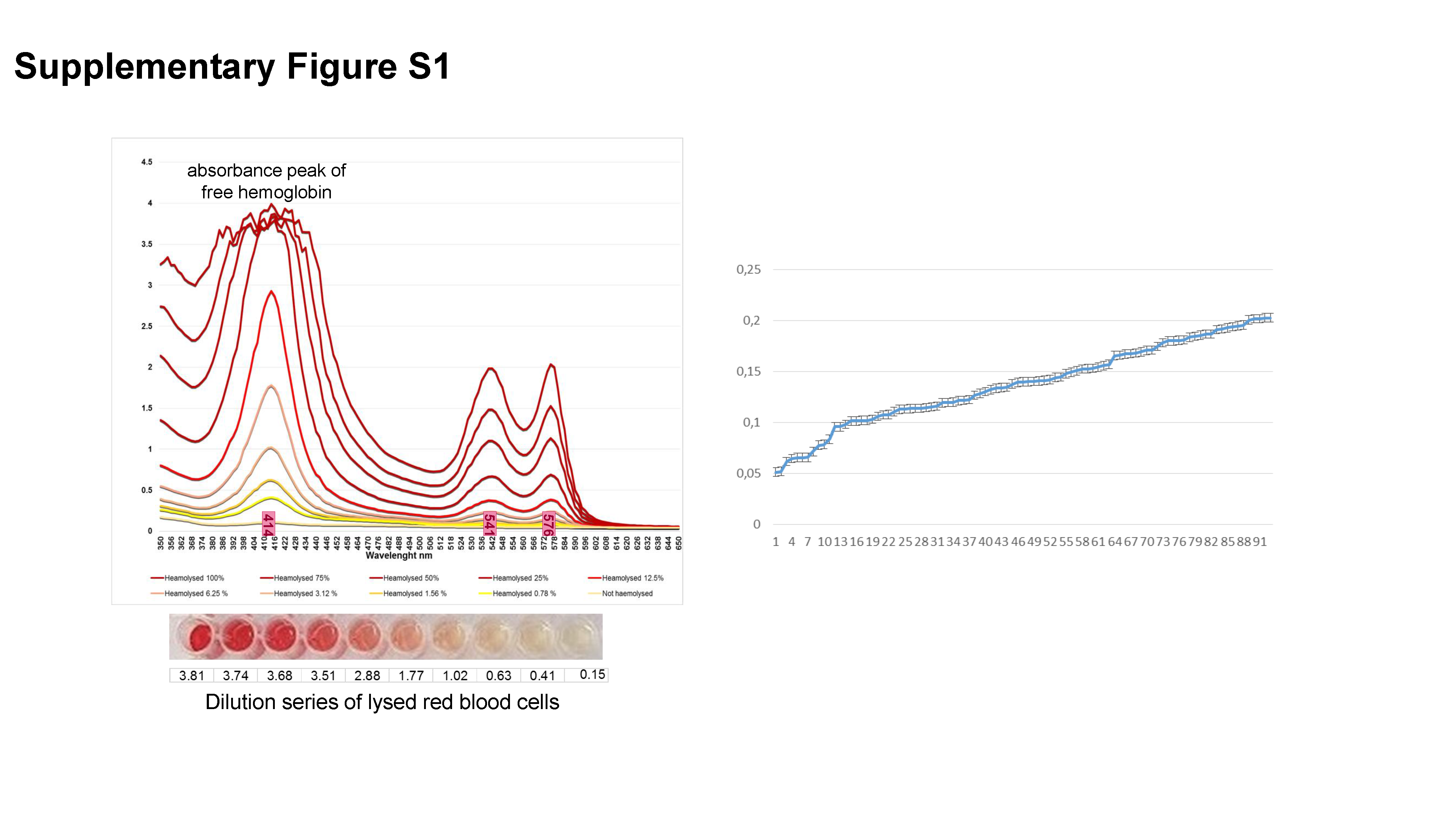

Supplement: Supplementary file 1 — Fig. S1. Levels of free hemoglobin measured in the plasma samples. [file MOL2-12-1935-s001.tif]

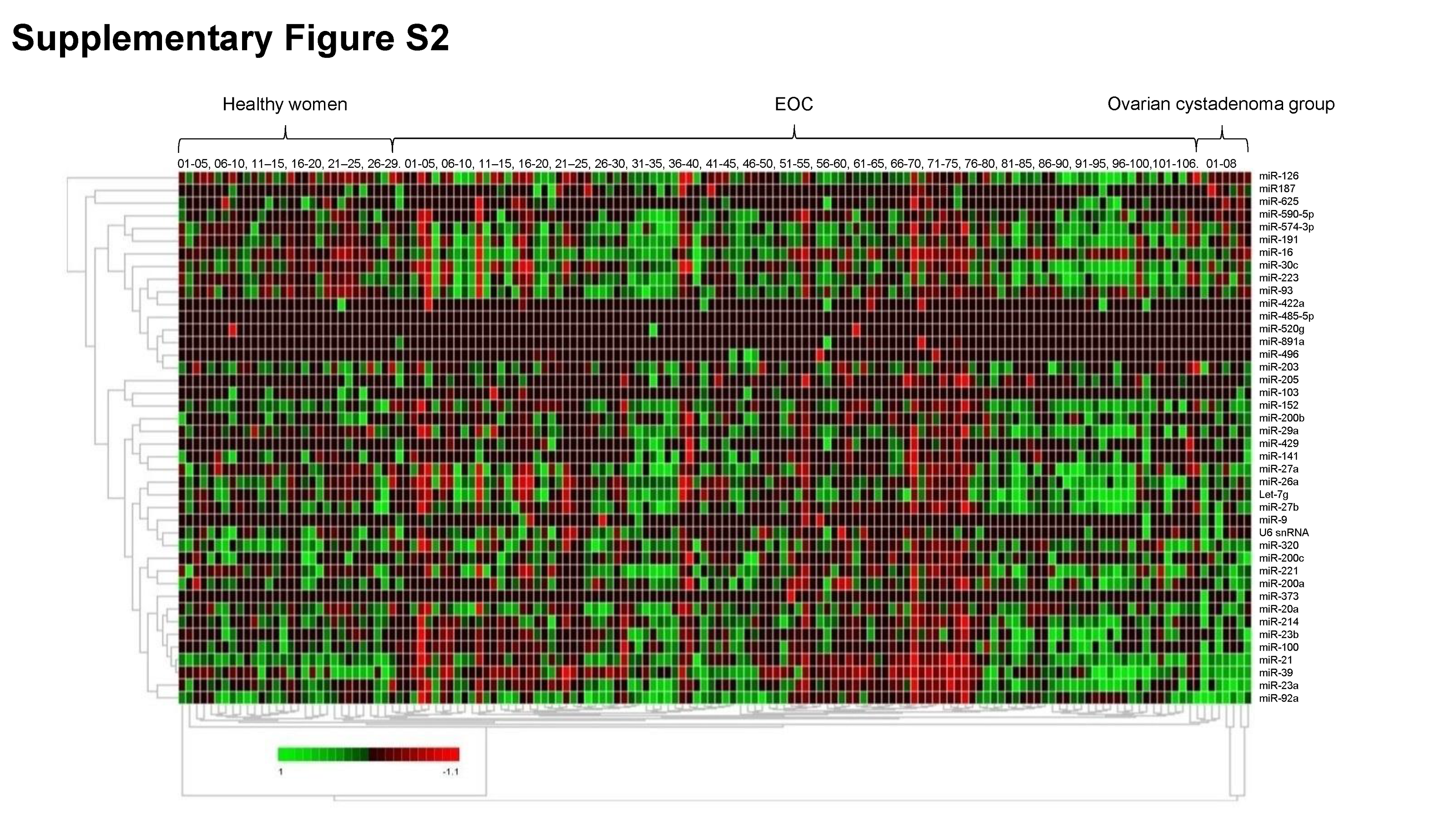

Supplement: Supplementary file 2 — Fig. S2. Hierarchical cluster of 48 exosomal miRNAs. [file MOL2-12-1935-s002.tif]

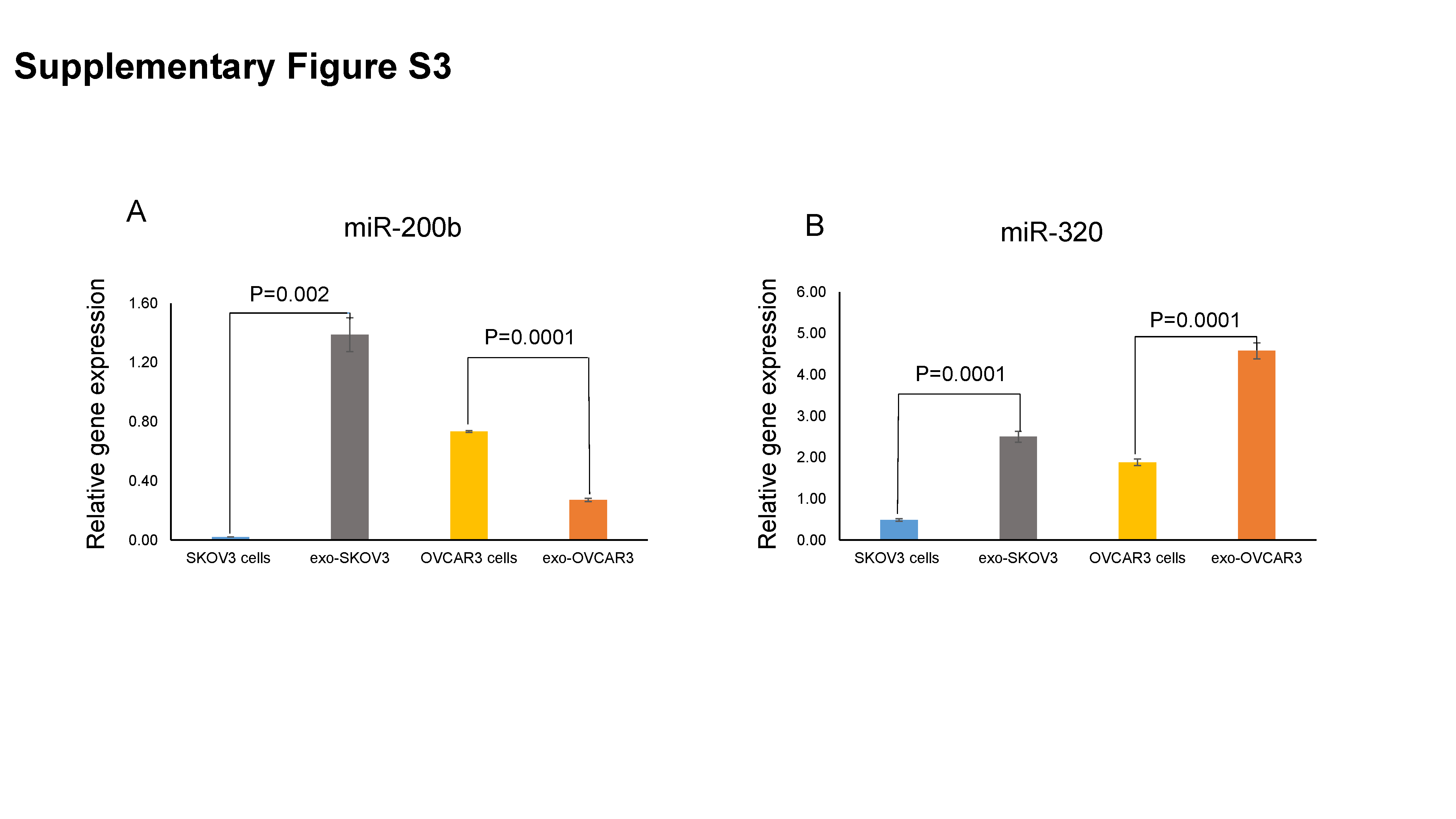

Supplement: Supplementary file 3 — Fig. S3. miR‐200b and miR‐320 levels in cell lines and their released exosomes. [file MOL2-12-1935-s003.tif]

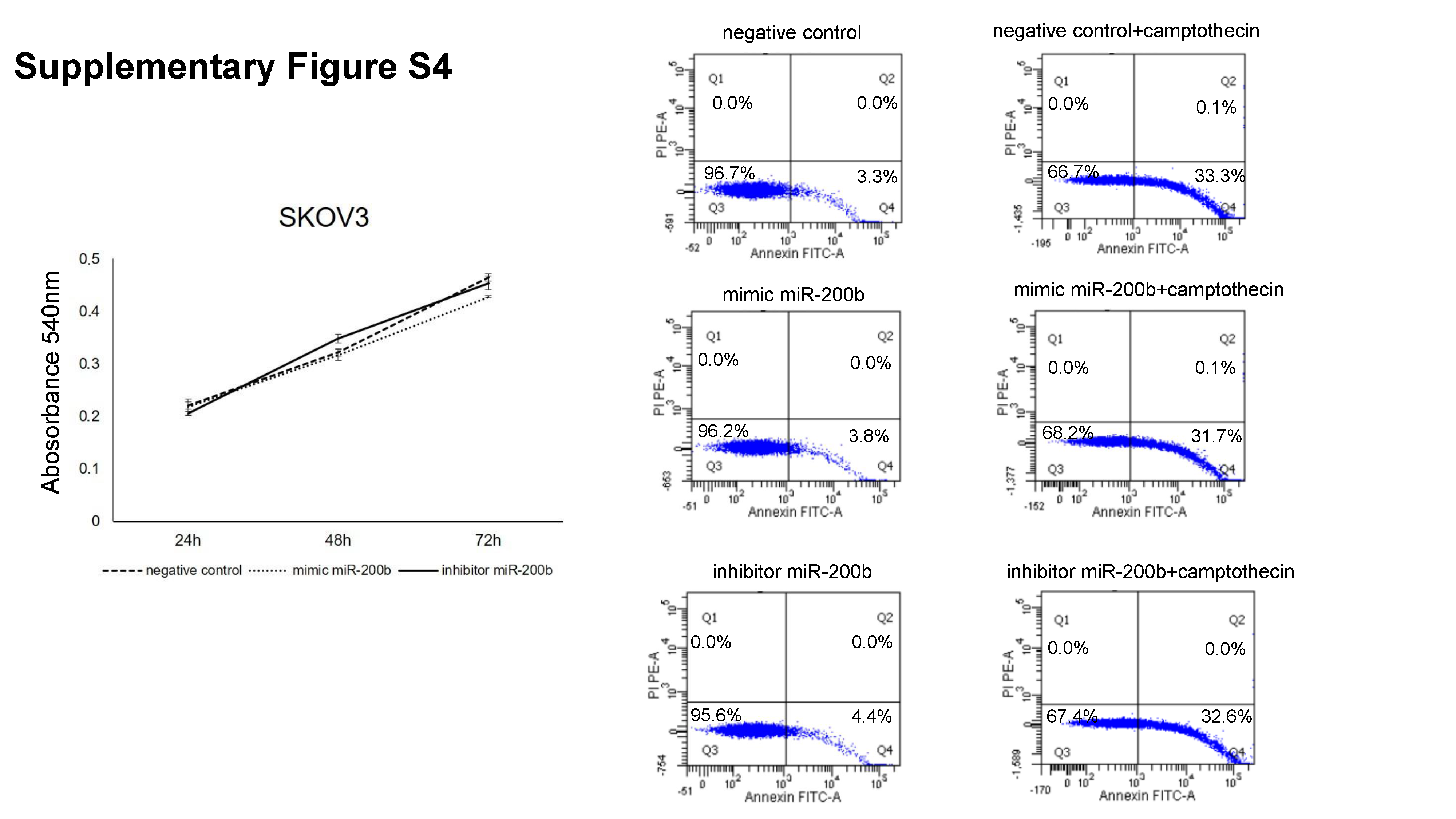

Supplement: Supplementary file 4 — Fig. S4. miR‐200b does not affect cell proliferation and apoptosis in SKOV3 cells. [file MOL2-12-1935-s004.tif]

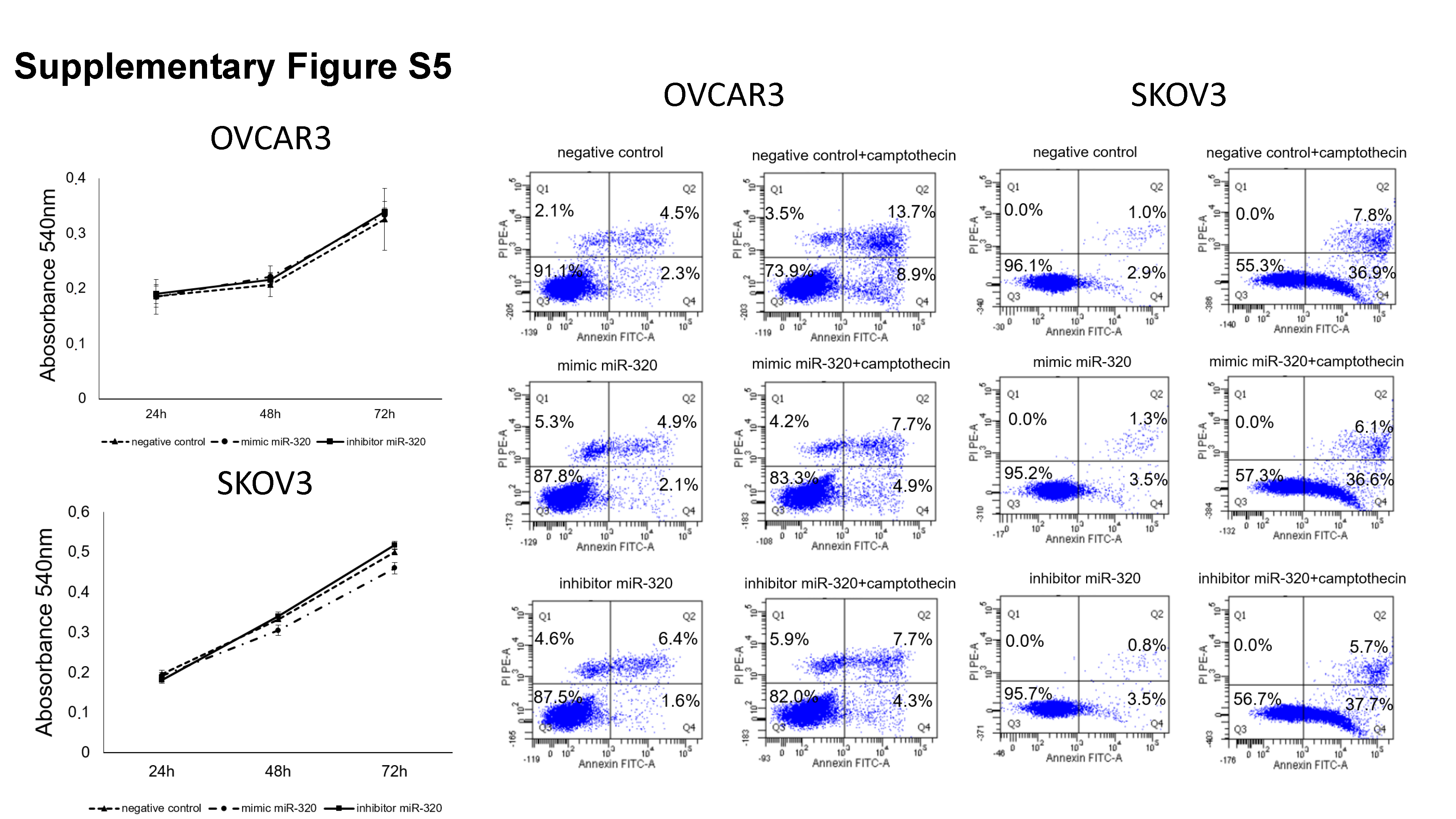

Supplement: Supplementary file 5 — Fig. S5. miR‐320 does not affect cell proliferation and apoptosis. [file MOL2-12-1935-s005.tif]
