# Supplementary material for: Exosomal microRNAs as tumor markers in epithelial ovarian cancer
Source: Mol Oncol. 2018 Oct 9;12(11):1935–48. doi: 10.1002/1878-0261.12371 (PMC6210043; doi:10.1002/1878-0261.12371)
Supplement: Supplementary file 6 [file MOL2-12-1935-s006.docx]

**Supplementary Figure Legends**

**Figure S1 Levels of free hemoglobin measured in the plasma samples**

Hemolysis was assessed by spectrophotometry at wavelengths from 350 to 650 nm. A dilution series of lysed red blood cells in plasma was prepared (below the chart). The degree of hemolysis was determined based on the optical density (OD) at 414 nm (absorbance peak of free hemoglobin, called Soret band), with additional peaks at 541 and 576 nm. Samples were classified as being hemolysed if the OD at 414 exceeded 0.25. The integrated curve of plasma samples comprises values from 0.05 to 0.20 indicating that the samples were non-hemolysed.

**Figure S2 Hierarchical cluster of 48 exosomal miRNAs**

The heat map is derived from data of the miRNA array cards which were performed using quantitative real-time PCR with assays for detection of 48 different miRNAs and using exosome samples from plasma of 106 EOC patients, 8 ovarian cystadenoma patients and 29 healthy women. The colored representation of samples and probes is ordered by their similarity. The red and green colors indicate that the ΔCq value is below (relatively high expression) and above (relatively low expression levels) the median of all ΔCq values in the study, respectively. On the right side: clustering of probes. The scale bar provides information on the degree of regulation.

**Figure S3 miR-200b and miR-320 levels in cell lines and their released exosomes**

The bar charts show the expression levels of miR-200b (A) and miR-320 (B) in OVCAR3 and SKOV3 cells along with those in their exosomes. Three independent experiments were performed. Error bars are presented as means ±SD. ANOVA Tukey’s HSD tests were used to determine the significance (p<0.05).

**Figure S4 miR-200b does not affect cell proliferation and apoptosis in SKOV3 cells**

SKOV3 cells were transiently transfected with a negative control, mimic or inhibitor of miR-200b. After 24, 48 and 72 hours, cell proliferation rate after overexpression and inhibition of miR-200b was measure at absorbance of 540 nm using MTT Cell Proliferation Assay. The standard deviations from triplicate experiments are indicated in the line chart. Transfected SKOV3 cells were additionally treated with the topoisomerase I inhibitor camptothecin to induce apoptosis, and analyzed on a FACS Canto II device. Cells were labeled with Annexin-V-FITC and propidium iodide for FACS analyses. Cell fragments only positive for propidium iodide can be found in the upper left corner (Q1). Late apoptotic as well as necrotic cells can be found in the upper right corner (Q2), since they are positive for Annexin and propidium iodide. Living cells are negative for Annexin and propidium iodide, and therefore, can be found in the lower left corner (Q3). Only early apoptotic cells are positive for Annexin, and located in the lower right corner (Q4). The size for each population (%) is given in the corresponding area.

**Figure S5 miR-320 does not affect cell proliferation and apoptosis**

OVCAR3 and SKOV3 cells were transiently transfected with a negative control, mimic or inhibitor of miR-320. After 24, 48 and 72 hours, cell proliferation rate after overexpression and inhibition of miR-320 was measure at absorbance of 540 nm using MTT Cell Proliferation Assay. The standard deviations from triplicate experiments are indicated in the line chart. Transfected OVCAR3 and SKOV3 cells were additionally treated with the topoisomerase I inhibitor camptothecin to induce apoptosis, and analyzed on a FACS Canto II device.
